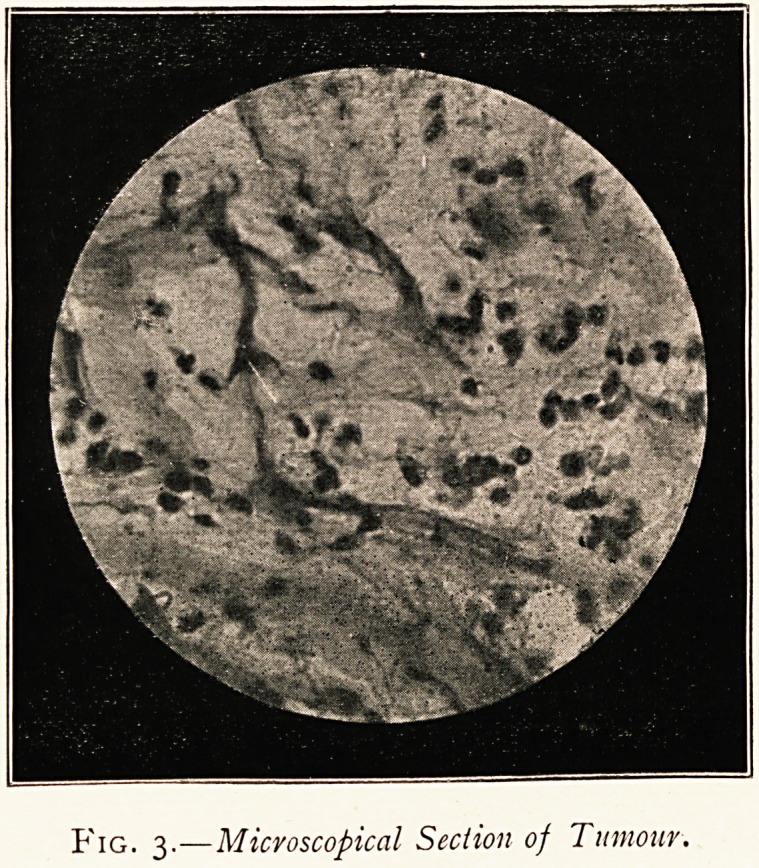# A Retrospect of a Third Series of Fifty Consecutive Intra-Abdominal Operations

**Published:** 1901-06

**Authors:** James Swain

**Affiliations:** Professor of Surgery at University College, Bristol; Assistant-Surgeon to the Bristol Royal Infirmary.


### A RETROSPECT OF A THIRD SERIES OF FIFTY CONSECUTIVE INTRA-ABDOMINAL OPERATIONS.

BY

JAMES SWAIN, M.S., M.D. Lond., F.R.C.S. Eng.,

*Professor of Surgery at University College, Bristol; Assistant-Surgeon to the Bristol Royal Infirmary.*

**(Continued from page 29.)**

---

*F.*—**Operations upon the Stomach.**—CASES 9 and 24.

**Gastrorraphy** for perforating **gastric ulcer** is most successful when performed within twelve hours of the perforation. After this period the mortality rapidly increases, so that it is highly desirable to proceed to operation as soon after the diagnosis is made as possible. In Case 9 operation was not performed till about forty-five hours after perforation, by which time a general septic peritonitis had occurred.

**Benign tumours of the pylorus** have not received much attention, partly because of their rarity, and partly because the pyloric obstruction which they cause is accompanied by symptoms resembling those produced by malignant neoplasms.

Benign tumours of the pylorus are associated with a degree of mobility which is more commonly found in new growths of the small intestine or omentum; and the lesson to be learnt from the following remarkable case, which I have described more fully elsewhere,[1] is that an innocent tumour of the pylorus should be suspected when there is a freely movable swelling in the epigastrium, with symptoms of pyloric stenosis and gastrectasis in a young adult.

CASE 24.—**Intra-pyloric Fibroma—Pylorectomy.**—The patient had suffered from attacks of indigestion, shown by vomiting and pain in the epigastrium after meals, since she was fifteen or sixteen years of age. Eight years ago she had "ulcer of the stomach" and passed black motions, though there was no hæmatemesis. About a month before I saw her she suffered from flatulence and unpleasant eructa-

[1] *Westminster Hosp. Rep.*, 1901, xii. 109.

tions, and noticed a hard lump in the abdomen about the size of a hen's egg. There was no pain, but she wasted rapidly and felt ill. For the past fortnight there had been attacks of vomiting. Emaciation was marked. Situated in the right umbilical region was a slight bulging caused by a firm, irregularly-shaped tumour nearly as large as the closed fist and having a nodular surface. Though usually situated just to the right of the umbilicus, the tumour was occasionally found in a different position (close under the right costal margin, &c.), and could be easily moved completely to the left of the umbilicus. With respiration the tumour moved downwards some two inches, and could be similarly displaced by manipulation, but its downward displacement seemed to be limited by some attachment above. There was resonance over the tumour, and gurgling could be felt by the hand and heard with the stethoscope, though by auscultatory percussion no definite connection between the tumour and the stomach could be made out. The vomit gave a negative reaction to Günzburg's reagent. A four-inch incision was made in the median line, about two-thirds of it being above and one-third below the umbilicus. Part of the stomach and duodenum were delivered through the parietal wound, and an incision made in the anterior wall of the stomach. The tumour was then found to be attached to the posterior part of the pylorus by a thick pedicle about one and a half inches in diameter, and the body of the tumour hung down in the stomach in front of the pylorus and evidently acted like a ball valve. The weight of the tumour had caused invagination of the adjacent parts of the stomach and duodenum. The stomach was dilated and contained a dark brownish-black fluid. By prolonging the incision in the anterior part of the stomach the growth was encircled, and then entirely removed with the adjacent parts of the stomach and duodenum. A cicatrised ulcer was seen on a small part of the pylorus, which was not excised. The duodenum was attached to the stomach by a double row of stitches and the abdomen closed without drainage. The patient only vomited about a drachm of watery fluid after operation. Nutrient enemata formed the sole support until the third day after operation, when feeding with peptonised milk and water gruel was commenced. The diet was gradually improved: bread and butter was allowed on the sixth and fish on the ninth day after operation. The patient got up about three weeks, and went home at the end of a month, after operation.

*Examination of Tumour.*—The portion removed weighed $4\frac{3}{4}$ ounces. The tumour sprang from a portion of the pylorus, and the adjacent parts of the stomach and duodenum had been drawn inwards by its weight, so that there was a cone-shaped recess covered by peritoneum at the base of the tumour. The tumour itself had an irregular shape and surface, and measured about $3\frac{1}{2}'' \times 2\frac{1}{2}'' \times 2''$ (Fig. 2). It was covered with reddish-grey mucous membrane, but the mucous membrane of the invaginated parts of the stomach and duodenum around its base was congested and of a purplish-red colour. It cut with a firm section, and the internal aspect was smooth and of a rather translucent and almost white colour. Microscopical examination showed it to be composed of bundles of fibrous tissue with loose cellular tissue (Fig. 3), but here and there it had a myxomatous appearance. The mucous membrane covering it was lined with columnar epithelium. I am indebted to Mr. James Taylor for the photo-micrograph and to Mr. Clayton for the photograph of the specimen itself.

Fig. 2.—*Anterior Aspect of Tumour.*

Fig. 3.—*Microscopical Section of Tumour.*

*G.*—**Operations for Diseases of the Intestines.**—CASES 1, 4, 7, 20, 25, 29, 34, 39, and 41.

These may be divided into—

**Colostomy.**—CASES 1, 7, 29, and 34.
**Colectomy.**—CASE 39.
**Operation for Volvulus.**—CASE 4.
**Enterorraphy.**—CASES 20 and 25.
**Operation for Perforation by Foreign Body.**—CASE 41.

All the cases of **colostomy** were performed for advanced malignant disease which did not admit of removal. Perhaps the insidious onset of malignant disease of the intestines is largely to blame for the comparative rarity of a sufficiently early operation to allow of a radical cure. But even if the time for excision is allowed to go by, it seems inexcusable that fæcal vomiting should be permitted to occur before relief is sought by colostomy—and this is by no means uncommon. To be successful, the operation should be performed before the patient is exhausted by the absorption of toxic products from his own intestines, so that the gut need not be opened at the time it is brought to the surface. When this obtains, colostomy is almost devoid of danger.

In the performance of the operation the muscles should be separated bluntly, and not cut. In this way I have frequently obtained a certain amount of sphincteric action around the artificial anus which is most helpful in the retention of the fæces, so that in some cases a motion occurs once daily without any leakage in the interval.

One of the most life-saving suggestions of Greig Smith was the performance of **enterectomy**, when intestinal obstruction is present, in three stages: firstly, fixation of the growth outside the body, and the immediate establishment of intestinal drainage; secondly, resection of the growth about ten days after; and, thirdly, closure of the artificial anus at a still later date. For some time past I have merged these last two stages into one, the ends of the divided intestine being approximated immediately after the growth is excised. This is a saving of time, and the operation is easier than in three stages, because the calibre of the two portions of the gut is less unequal and there is no marked "spur" to contend with.

Recovery after operation for intestinal obstruction at the age of 78 years is extremely rare, and it is even more uncommon for that recovery to be associated with the removal of a malignant growth by **colectomy** (Case 39).

CASE 39.—**Malignant Stenosis of the Descending Colon—Colectomy.**— The patient had had several attacks of obstruction during the preceding twelve months. For ten days before operation there had been symptoms of incomplete obstruction with vomiting, and for twenty-four hours before operation there was complete obstruction with gradually increasing abdominal distension, pain, and vomiting. A coil of the descending colon with the malignant growth at the apex was fixed over a glass rod outside an oblique incision in the left flank, and a medium-sized Paul's tube was placed in the upper end of the loop, by which a pint of liquid fæces and much flatus soon came away. The Paul's tube was cut away on the third day, and the patient allowed to get up on the tenth day after operation. On the seventeenth day after operation the colon was completely cut across above and below the growth, and the latter thereby freely excised. The two ends of the gut were then immediately united by a double layer of sutures. A fæcal fistula formed, and an unsuccessful attempt was made to close it. This still persists, as the advanced age (78 years) of the patient renders further operation undesirable. The portion of gut removed was about three inches long on the convex surface. The growth—which was a columnar epithelioma—occupied about one and a half inches of the whole circumference of the gut, and had almost completely obliterated the lumen.

It is not often a patient is subjected to two operations for intestinal obstruction. The following case of **volvulus** is interesting because it shows what good union may be effected by the Murphy button, an instrument which for several reasons I do not use if intestinal anastomosis can be safely effected by immediate suture.

CASE 4.—**Volvulus from Adhesions after the Use of the Murphy Button.**—Six years ago the patient had suffered from a strangulated femoral hernia, resulting in gangrene of the gut. Enterectomy was then performed, and the two ends of the gut were approximated by a Murphy button, the operation being carried out through a median incision. At the time of the second operation the patient was suffering from acute intestinal obstruction. On cutting through the cicatrix between the umbilicus and pubes the underlying bowel was wounded, because it was firmly adherent to the cicatrix. The peritoneal cavity was therefore entered above the cicatrix, and it was then found that a portion of ileum was adherent along the whole length of the cicatrix over a linear distance of about two and a half inches. On these adhesions as an axis the bowel had become twisted, so that the proximal and distal portions strangled each other. The intestine above the seat of volvulus was distended; below it was contracted. The gut was freed from adhesions and untwisted. After allowing the intestine to empty itself through the hole which had been accidentally made, the aperture was closed with Lembert sutures. The parietal wound was closed without drainage. The bowels acted during the same night, and the patient made an uninterrupted recovery. The cicatrix in the bowel where the Murphy button had been used and the part where the mesentery had been sewn were rather whiter than normal. The line of union of the gut was quite firm, and there was practically no narrowing of the lumen.

The following case puzzled me greatly. Whether it began as a burst tubo-ovarian abscess, which subsequently discharged into the jejunum, or whether the jejunal perforation was the primary lesion, I am quite unable to tell. In any event, recovery after operation for **jejunal fistula** is sufficiently rare to justify an account of the facts :—

CASE 20.—**Jejunal Fistula—Enterorraphy.**—The patient had had seven confinements, the last being three weeks before she came under my observation. During the last pregnancy the abdomen was said to have been more distended than usual, and the distension, instead of diminishing after labour, gradually increased and the bowels became very tender. When first seen the abdomen was as large as at a full-term pregnancy, and appeared to be occupied by a cystic swelling in the lower part. There was slight fever, and the general condition was one of some gravity. A median incision was made between the umbilicus and pubes, and a cavity opened containing six pints of a dark reddish offensive fluid. The wall of the cavity appeared like the inside of an universally adherent cyst, and the general peritoneal cavity was entirely shut off. Two large drainage tubes four inches long were placed in the wound, which continued to discharge a considerable amount of offensive fluid. Five days later the discharge was found to be acid in reaction, and contained bile acids and crystals like uric acid and sodium glycocholate, but no mineral acid. This caused an extensive redness and excoriation of the skin of the abdomen around the wound, exactly as sometimes occurs in the neighbourhood of a gastric fistula. Grape-stones, portions of orange and other indigestable articles of diet, began to be discharged through the wound, but the cavity gradually contracted down to a small fistula, which finally closed about seven weeks after operation. This closure was, however, only temporary, for directly the discharge ceased the temperature rose, and the wound again began to discharge nine days later. This discharge was more green than before, and its persistence from the fistulous opening led to the second operation, about nine weeks after the first operation. The fistulous opening (one and a-half inches below the umbilicus) in the parietes was slit up, and an incision about eight inches long was made from a point three inches above the umbilicus downwards towards the pubes. There was much matting of the intestines and omentum in the neighbourhood of the fistula, but the mass was gradually unravelled to allow of evisceration. A careful search for the fistula was made in the stomach and duodenum because of the acid character of the discharge, but no evidence of ulcer was found. A perforation the size of a large quill was afterwards discovered in the upper jejunum (?), which was adherent to the right side of the incision in the parietes. Considerable matting was found in the neigh bourhood of the left ovary, and the peritoneum generally was much thickened. The perforation in the intestine was sutured with Halsted's stitches, the abdominal contents which had been turned out were returned, and a large tube was placed across the abdominal cavity—one end emerging at the mid-line and another at a counter opening in the right flank. A second tube was placed in Douglas's pouch. Convalescence was protracted, but the patient made a good recovery.

In cases of **abdominal contusion** it is not always easy to say whether there is a visceral injury or not. In most instances in which the shock is prolonged, cœliotomy is called for. For the diagnosis and treatment of these injuries, I would refer to my article in the *Encyclopædia Medica*[1]. In the following case the typical symptoms of marked shock, accompanied by evidence of free gas in the peritoneal cavity and persistent vomiting, led to a correct diagnosis of ruptured intestine.

CASE 25.—**Rupture of Small Intestine—Enterorraphy.**—The patient was thrown from his bicycle, and was supposed to have been struck in the abdomen by the shaft of a carriage. He complained of great abdominal pain. Vomiting set in an hour after the accident and persisted, and the shock gradually became more marked. At the time of examination he was very pale and restless, and complained of intense pain in the abdomen below and to the left of the umbilicus and in each supra-clavicular fossa. Pulse 140 per minute, small and compressible; respiration almost entirely thoracic; abdomen rigid and not distended. There was resonance all over the usual area of liver dulness. After a hypodermic injection of strychnine and a rectal injection of brandy the abdomen was opened, nineteen hours after the accident, above the umbilicus. On incising the peritoneum free gas escaped, followed by a large quantity of dark liquid blood and clots, and large clots were scooped out by the hand. The omentum and some coils of intestine were bruised and congested. A portion of gut, which was probably the upper jejunum, had a large rent on the convex border, the hole being as large as a penny-piece and the edges being ragged, bruised and bleeding. The aperture in the gut was quickly closed by a series of Halsted's quilt sutures placed longitudinally to the gut, so that the line of union would be transverse to the long axis of the gut and prevent stenosis. The abdomen was sponged out and a drainage tube inserted before closing the parietal wound. The stomach and liver were uninjured. The condition of the patient was desperate; he rallied at first, but died suddenly soon after operation.

Wonderful recoveries sometimes occur from **perforation of the intestine** by foreign bodies which have been swallowed, but Case 41 shows what serious results may follow the swallowing of a sharp-pointed body like a pin:—

CASE 41.—**Perforation of Intestine by a Pin—Peritonitis.**—Three weeks before I saw him the boy had been to a school treat, and had complained of abdominal pain in the neighbourhood of the cæcum

[1] *Encyclopædia Medica*, vol. i., 1899, p. 5.

ever since. The boy looked very ill; temperature, 100°; pulse, 116°. A large hard mass occupied the right half of the abdomen. An incision into this released some ounces of pus, and deep down towards the pelvis I extracted a common pin (black from decomposition) from the centre of a dense inflammatory mass. The boy never did well, in spite of further operations to ensure thorough drainage. He developed pyæmic symptoms, and a culture from the pus showed a mixed infection of streptococci and B. coli and pyocyaneus (Prof. Kent). Antistreptococcic serum was given in large doses, but the patient is now (about three months after the extraction of the pin) in an apparently dying condition.[1]

*H.*—**Operations for Appendicitis.**—CASES 2, 3, 8, 11, 15, 23, 26, 28, 37, 38, 40, 42, 44, 46, 47, and 48.

The limitations of space prevent me from dilating upon so interesting a subject as when to operate in appendicitis. I have recently discussed the whole matter somewhat fully by a consideration of fifty consecutive operations for appendicitis.[2] Suffice it to say that the conclusion I have formed is that it is far more rational, and equally safe, to treat each case on its merits, rather than to indulge in such wholesale surgery as would necessarily result from the adoption of any such hard and fast rule as that of operating upon all cases that are not improving in forty-eight hours from the commencement of the attack.

Whenever it is necessary to remove an appendix, I generally prefer to wait until an attack—if it exists—is over, and I have never lost a case which has been operated upon in an "interval." In the above series, I performed **appendicectomy** in Cases 11, 15, 23, 26, 28, 37, 38, 42, 44, 46, 47, and 48 without a single death, but most of them were "interval" cases.

Of the value of **abdomino-vaginal drainage** in cases of extensive pelvic suppuration, whether due to appendicitis or not, I have spoken of in a previous paper; but whether this method of drainage is adopted or not, the head of the bed should be raised in all cases associated with a wide-spread septic peritonitis, for by this means there is a tendency for the inflammation to be confined to the pelvis where its effects, as Dr. Fowler[3]

[1] Death occurred a fortnight after the above account was written. The *post-mortem* examination disclosed extensive actinomycosis, the organism possibly being taken into the system at the time the pin was swallowed.

[2] *Westminster Hosp. Rep.*, 1901, xii. 49—64.

[3] *Med. Rec.*, 1900, lvii. 617.

points out, are less serious than when the upper part of the peritoneal cavity is affected.

The differential **diagnosis** of acute inflammation of the uterine appendages and appendicitis is frequently very difficult, and in a complicated case like the following it is practically impossible to be certain of the primary focus of the disease even after operation:—

CASE 37.—**Salpingo-oöphoritis—Appendicitis.—Salpingo-oöphorectomy—Appendicectomy.**—The patient had had typhoid fever ten months before, and this was followed by an inflammatory attack which was supposed to be due to appendicitis. Since then there had been more or less constant pain in the right side of the abdomen, and three weeks before I saw her she again had symptoms of peritonitis beginning in the right iliac fossa with a temperature of 103°. For the past week the temperature had been normal. Menstruation had been regular and painless. At the time of examination by bi-manual palpation, a swelling was felt in the right iliac region, spreading out half-way along Poupart's ligament from the middle line, and reaching upwards to a point about two-thirds of the distance from the middle of Poupart's ligament to the umbilicus. Pressure over McBurney's spot did not elicit tenderness. The uterus was fixed, and its fundus apparently reached to one and a half inches above the pubes. The right vaginal fornix was depressed by a swelling which appeared to be in the broad ligament close to the uterus, and cystic in nature. The left broad ligament was densely infiltrated, but no fluctuation was present. The patient was put in the Trendelenburg posture, and a median incision four inches long was made between the umbilicus and pubes. All the structures in the pelvis were found to be densely matted. On the right of the uterus (which could not be defined at this stage because of adhesions) was a fluctuating swelling occupying the right broad ligament. Above and to the inner side of this were the cæcum and appendix, densely adherent and the latter bent on itself. The right broad ligament was opened, and an attempt made to enucleate the fluctuating swelling. The bleeding was free, and owing to the density of the adhesion the abscess sac—for such it was—burst, and a fœtid pus escaped. The patient was at once put down from the Trendelenburg posture, and the intestines walled off with iodoform gauze. As pus was escaping, the enucleation was rapidly proceeded with, and a mass the size of an orange was delivered and cut away, after a pedicle composed of part of the Fallopian tube and broad ligament had been tied off. The cæcum and appendix were freed from their remaining adhesions, and then, for the first time, the uterus was definable. The left ovary and tube were embedded in a mass of dense adhesions, and in trying to separate them another abscess cavity was opened in the left broad ligament. This abscess sac was enucleated, and the pedicle formed by the Fallopian tube and ovary tied and cut off, as on the right side; but in so doing there was considerable tearing of the peritoneal surface of the bladder over an area of about two inches by one inch. The appendix (which was thickened and inflamed, and contained fæcal matter) was then removed close to the cæcum, and the stump sequestered. The rent in the peritoneal covering of the bladder was sewn up with interrupted silk sutures, the pelvis sponged out, and gauze and tube drainage placed behind the uterus. The parietal incision was closed with silkworm gut, and the head of the bed raised to confine the exudation to the pelvis. The gauze and tube were removed on the second day, recovery was rapid and complete, and the patient was able to travel within four weeks of the operation.

A few of the more interesting features of some of the other cases may be briefly stated:—

CASE 2.—Perforation had apparently occurred forty-eight days before operation, and the abdomen contained a large quantity of liquid fæces shut off from the peritoneal cavity. Fæces continued to be discharged through the abdominal wound for more than a fortnight before death occurred.

CASE 8.—There was a large abscess cavity, containing about a pint of fœtid pus, occupying the whole sacral hollow and true pelvis—the uterus and appendages standing out in the middle of the cavity. There were no limiting adhesions. Successful treatment by abdomino-vaginal drainage, but appendicectomy had to be performed subsequently for a recurrence of attacks.

CASE 15.—The inflamed appendix was as large as the thumb, and was with difficulty found in the midst of an extensive fibrinous exudate.

CASE 28.—Resembled Case 37, which has been described at length above. Abscesses existed in connection with the Fallopian tubes, and a double salpingo-oöphorectomy was performed in addition to appendicectomy. Abdomino-vaginal drainage. A fæcal fistula followed.

CASE 38.—There was a gangrenous and perforated appendix, which was removed within twelve hours of the perforation.

*J*.—**Exploratory Operations.**—CASES 10, 13, 31, 33, 35, 36, and 50.

In the present state of abdominal surgery it is not possible to avoid opening the abdomen for the purpose of making an exact diagnosis of the nature and extent of the disease in a certain number of cases. An exploratory operation is generally a perfectly safe procedure, and it is gratifying therefore to know that no harm was done to any of the patients in whom it was employed.

Cases 33, 35, 36, and 50 were associated with malignant disease, and it is especially in this class of case that it is often impossible to tell whether the affection is capable of removal or not until its extent is made known by means of an explanatory cœliotomy. The regret in most of these cases is that operation was not undertaken at an earlier period of the disease, at a time when surgery holds out a better prospect of cure.

## Figures and Tables

**Fig. 2. f1:**
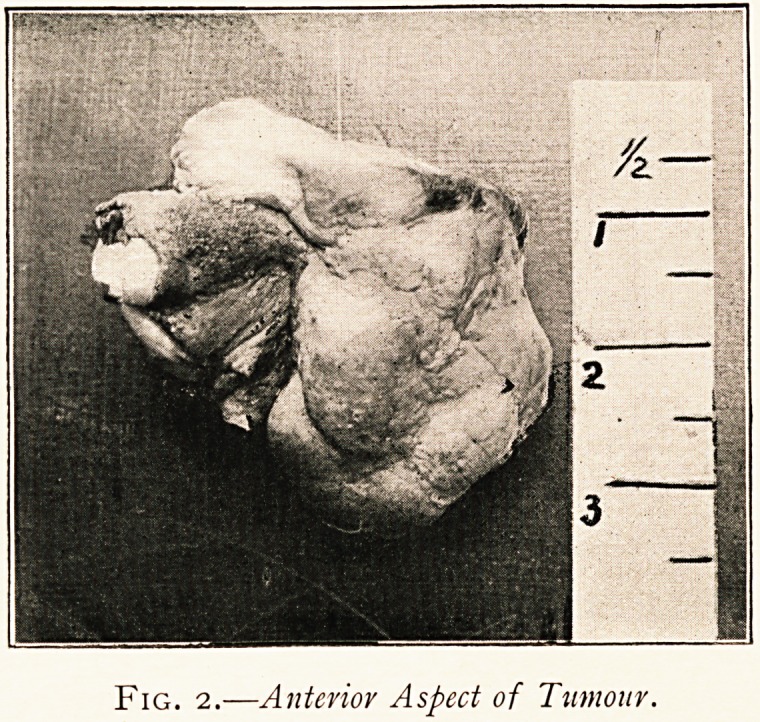


**Fig. 3. f2:**